# Indoor Employment as a Factor Associated with Vitamin D Levels in Obstructive Sleep Apnoea Syndrome

**DOI:** 10.3390/medicina62020388

**Published:** 2026-02-16

**Authors:** Evangelia Nena, Kostas Archontogeorgis, Maria Katsaouni, Konstantina Chadia, Athanasios Voulgaris, Paschalis Steiropoulos

**Affiliations:** 1 Laboratory of Social Medicine, Medical School, Democritus University of Thrace, 68100 Alexandroupolis, Greece; enena@med.duth.gr; 2 MSc Program in Sleep Medicine, Medical School, Democritus University of Thrace, 68100 Alexandroupolis, Greecethanasisvoul@hotmail.com (A.V.); 3 Department of Pneumonology, Medical School, Democritus University of Thrace, 68100 Alexandroupolis, Greece; con_chadia@yahoo.gr

**Keywords:** environment, obstructive sleep apnoea syndrome, occupation, 25-hydroxyvitamin D

## Abstract

*Background and Objectives*: Variations in serum 25-hydroxyvitamin D [25(OH)D] concentrations have been described among individuals working in different settings. Moreover, published evidence suggests an association between Obstructive Sleep Apnea Syndrome (OSAS) and 25(OH)D insufficiency. The aim of this study was to assess the association between certain exposures in the occupational environment and 25(OH)D levels, in relation to OSAS severity. *Materials and Methods*: For a period of 12 months, 25(OH)D serum levels were assessed in subjects consecutively investigated for OSAS. These were divided into group A (control subjects working indoors), group B (control subjects working outdoors), group C (OSAS patients working indoors) and group D (OSAS patients working outdoors). *Results*: A total of 189 subjects were included (155 males and 34 females), comprising 129 OSAS patients and 60 non-apnoeic controls. Serum 25(OH)D levels were significantly higher in group B compared to the other groups (32 ± 13 vs. 23 ± 7 ng/mL for group A, *p* = 0.001; vs. 15 ± 8 ng/mL for group C, *p* < 0.001; and vs. 23 ± 9 ng/mL for group D, *p* < 0.001). Additionally, serum 25(OH)D levels were higher in group A compared with group C (23 ± 7 vs. 15 ± 8 ng/mL, *p* = 0.001), while no significant difference was observed between groups A and D (*p* > 0.05). Regression analysis revealed that the apnoea–hypopnoea index (β = 0.02, OR = 1.02, 95% CI: 1.00–1.04, *p* = 0.02) and indoor occupation (β = 1.03, OR = 2.80, 95% CI: 1.17–6.70, *p* = 0.02) were independently associated with vitamin D insufficiency. *Conclusions*: Serum 25(OH)D levels are decreased in OSAS patients working indoors. Thus, the working environment should also be considered in the overall assessment of 25(OH)D status in OSAS patients.

## 1. Introduction

Obstructive Sleep Apnoea Syndrome (OSAS) is a sleep-related breathing disorder, characterized by recurrent episodes of partial or complete upper airway obstruction that lead to nocturnal hypoxia, sleep fragmentation, and excessive daytime sleepiness, with an increasing prevalence—driven by rising obesity rates—thereby posing a major public health burden [1,2]. OSAS has been linked to adverse cardiovascular events and an increased risk of metabolic disorders [3,4]. Moreover, untreated OSAS is associated with an elevated risk of traffic accidents and adverse effects on quality of life and work productivity [5,6].

Vitamin D [25(OH)D] is a fat-soluble vitamin primarily synthesized in the skin following sunlight exposure or acquired through diet, and it plays an essential role in calcium homeostasis [7]. In view of its relatively prolonged half-life and stable hepatic production, serum 25-hydroxy-vitamin D [25(OH)D] is the recommended indicator of overall 25(OH)D status; serum concentrations < 20 ng/mL are considered detrimental to bone health [8]. Vitamin D also acts as a neuroendocrine regulator involved in sleep regulation. Serum 25(OH)D levels have been positively correlated with key sleep parameters, including sleep quality, duration, and efficiency. Vitamin D may influence sleep through several interconnected molecular pathways, such as modulation of neurotransmitter systems, maintenance of circadian rhythm stability, and regulation of neuroimmune processes [9]. These effects are mediated by the widespread expression of vitamin D receptors in brain regions integral to sleep–wake regulation, allowing vitamin D to influence complex physiological processes underlying sleep. Proposed mechanisms include the regulation of neurotransmitter metabolism, preservation of neural plasticity, modulation of inflammatory cytokines and oxidative stress, and potential interactions with melatonin secretion pathways [9]. Vitamin D deficiency has been linked to a significantly higher risk of sleep disorders, poor sleep quality, shorter sleep duration, and excessive daytime sleepiness [10]. In contrast, existing evidence highlights the positive impact of vitamin D supplementation on sleep quality, emphasizing its important role in overall sleep health [11].

With prevalence rates of up to 40.4% recorded throughout European populations, 25(OH)D deficiency is a major global health concern [12]. Vitamin D deficiency remains highly prevalent across Europe and worldwide, as documented in more recent updates and large-scale syntheses, while earlier Europe-focused studies reported comparable concerns [12,13]. Insufficient sunlight exposure, inadequate dietary intake, and malabsorption are among the principal contributors to this deficiency [14]. Moreover, evening or night shift work, as well as indoor occupations, have been linked to an increased risk of 25(OH)D deficiency [15,16].

A growing body of evidence suggests an association between OSAS and 25(OH)D insufficiency, which appears to worsen with increasing sleep apnoea severity [17]. Although this was partially explained by shared, overlapping risk factors that predispose to both OSAS and hypovitaminosis D (e.g., obesity, older age), results from a meta-analysis challenged this assumption, indicating that additional pathogenic mechanisms, such as chronic low-grade inflammation or excessive daytime sleepiness, may also play a role [18,19]. However, a causal relationship between the two conditions cannot be excluded [20].

Accordingly, this study aimed to compare 25(OH)D serum levels between OSAS patients and non-apnoeic controls across different occupational groups, and to investigate the association between the working environment and 25(OH)D insufficiency in this population.

## 2. Materials and Methods

### 2.1. Patients

Subjects referred to the Sleep Unit of our institution for symptoms suggestive of sleep disordered breathing, who were actively employed in various occupations and provided written informed consent, were consecutively recruited over a 12-month period. This study was conducted in accordance with the Helsinki Declaration of Human Rights [21]. The study protocol was approved by the Institutional Ethics Committee.

Exclusion criteria were inability or unwillingness to participate; central sleep apnoea syndrome; conditions affecting calcium, phosphorus, and 25(OH)D metabolism and/or absorption; severe heart failure; inflammatory diseases; cancer; chronic liver or renal disease; osteoporosis; sleep disorders other than OSAS; 25(OH)D supplementation and/or corticosteroid therapy.

Detailed information on medical history, current medication use, with emphasis on 25(OH)D supplements, and smoking status was recorded. Information on typical occupational activities and work schedule, dress habits, and use of sunscreens or other protective measures was also obtained. Indoor occupations primarily comprised office-based and administrative work, whereas outdoor occupations mainly included field-based activities, such as agricultural work, construction work, and military service.

All participants underwent a comprehensive physical examination, during which height, weight, neck circumference, hip circumference, and waist circumference were measured according to a standardized protocol. Body mass index (BMI) was calculated using the following formula: BMI = weight (kg)/height^2^ (m^2^).

Daytime sleepiness was evaluated using the validated Greek version of the Epworth Sleepiness Scale (ESS) [22]. The scale comprises eight items describing common daily situations. Respondents rate their likelihood of falling asleep on a scale of 0–3 for each item. The maximum total score is 24, and scores >10 indicate excessive daytime sleepiness.

Pulmonary function tests, arterial blood gas analysis, and a 12-lead electrocardiogram were also performed to exclude underlying pulmonary and cardiovascular disease.

### 2.2. Polysomnography (PSG)

Overnight polysomnography (PSG) was performed using a standard clinical montage. All PSG studies were acquired using validated clinical systems and manually scored by trained sleep technologists, following AASM criteria for sleep staging and respiratory event scoring [23,24].

### 2.3. Blood Samples and Measurements

Venous blood samples were collected from all participants shortly after completion of the PSG. Blood was drawn after at least 8 h of fasting, immediately centrifuged (3000 rpm for 10 min), and the serum was separated and stored at −80 °C until analysis. Biochemical analyses were performed using an automated analyzer and included measurements of serum glucose, total cholesterol, triglycerides, high-density lipoprotein cholesterol (HDL-C), low-density lipoprotein cholesterol (LDL-C), creatinine, alanine aminotransferase (ALT), aspartate aminotransferase (AST), and C-reactive protein (CRP). Serum 25(OH)D concentrations were measured using a commercially available radioimmunoassay kit in accordance with the manufacturer’s instructions (DiaSorin, Stillwater, MN, USA). Serum 25(OH)D levels < 20 ng/mL were considered indicative of 25(OH)D insufficiency [25].

### 2.4. Statistical Analysis

Analysis was performed using the Statistical Package for Social Sciences version 23 (IBM SPSS, Armonk, NY, USA). Normality of distribution was assessed using the Kolmogorov–Smirnov test. Categorical variables were expressed as frequencies, continuous variables with normal distribution as mean ± standard deviation, and skewed variables as median (25th–75th percentile). Differences in categorical variables were evaluated using the chi-squared test. Correlations were examined using Pearson’s correlation coefficient. Group comparisons were conducted using one-way ANOVA or the Kruskal–Wallis test, as appropriate, based on data distribution. Post hoc comparisons for parametric data were performed using Tukey’s test, whereas Dunn’s multiple comparison test was applied for non-parametric variables. A binary logistic regression model was used to assess the possible association between 25(OH)D insufficiency and occupation after adjusting for anthropometric, demographic, and sleep parameters. Statistical significance was defined as a two-tailed *p* < 0.05.

## 3. Results

In total 189 subjects (155 males and 34 females; mean age 48 ± 10 years) participated in the study, including 129 OSAS patients (12 with mild, 17 with moderate and 100 with severe OSAS) and 60 non-apneic controls. Their characteristics are displayed in Table 1.

### 3.1. Correlations Between 25(OH)D Levels and Sleep Characteristics

Serum levels of 25(OH)D were positively correlated with REM sleep stage duration (r = 0.15, *p* = 0.04), as well as with average (r = 0.34, *p* < 0.001) and minimum (r = 0.31, *p* < 0.001) oxyhaemoglobin saturation during sleep. Negative correlations were observed with ESS score (r = −0.18, *p* = 0.02), sleep stage 1 duration (r = −0.15, *p* = 0.04), time spent with oxyhaemoglobin saturation < 90% (r = −0.29, *p* < 0.001), the arousal index (r = −0.33, *p* < 0.001), body mass index (r = −0.17, *p* = 0.02), and AHI (r = −0.45, *p* < 0.001).

OSAS patients and non-apneic controls were divided according to their occupation into the following smaller groups: group A (subjects working indoors with no OSAS, n = 30: 21 males and 9 females), group B (subjects working outdoors with no OSAS, n = 30: 24 males and 6 females), group C (OSAS patients working indoors, n = 64: 55 males and 9 females) and group D (OSAS patients working outdoors, n = 65: 55 males and 10 females). The characteristics of each group are displayed in Table 2 (anthropometric/demographic). The comparison of sleep parameters between groups is presented in Table 3. 

As shown in Table 2, patients with OSAS (groups C and D) exhibited significantly higher body mass index values compared with non-OSAS subjects (groups A and B; *p* = 0.001).

No significant differences were observed between these four groups in terms of indices of renal and liver function or lipid profile. Patients in group D exhibited worse respiratory function compared to those in group A. All laboratory results are presented in Table 4.

As seen in Table 5, serum 25(OH)D levels were highest in group B compared with all other groups (32.2 ± 13 vs. 22.9 ± 6.7 ng/mL for group A, *p* = 0.001, vs. 15.2 ± 7.6 ng/mL for group C, *p* < 0.001 vs. 22.9 ± 9.3 ng/mL for group D, *p* < 0.001). Additionally, serum 25(OH)D levels were increased in group A compared with group C (22.9 ± 6.7 vs. 15.2 ± 7.6 ng/mL, respectively, *p* = 0.001) and were similar between groups A and D.

### 3.2. Predictive Factors for 25(OH)D Results—Binary Logistic Regression Analysis

Binary logistic regression analysis revealed that the Apnoea–Hypopnoea Index (AHI) (β = 0.023, OR: 1.023, 95% CI: 1.003–1.043, *p* = 0.02) and indoor occupation (β = 1.03, OR = 2.80, 95% CI: 1.17–6.70, *p* = 0.02) were associated with 25(OH)D insufficiency [defined as 25(OH)D serum levels < 20 ng/mL], independently of age, sex, BMI, WHR, hypoxia indices during sleep, arousal index and ESS.

## 4. Discussion

We identified AHI and working indoors as independent predictors for 25(OH)D insufficiency. The relationship between 25(OH)D insufficiency and OSAS has been previously explored, but findings have been inconsistent [18,26,27]. In the present study, serum 25(OH)D concentrations were significantly predicted by AHI, consistent with the findings of a recent systematic review and meta-analysis that indicated an inverse association between serum 25(OH)D levels and OSAS severity in patients without comorbid conditions [27].

Similarly, according to another meta-analysis, patients with OSAS have lower circulating serum 25(OH)D levels and a higher prevalence of 25(OH)D deficiency compared with patients without OSAS, with the largest discrepancies observed among patients with moderate-to-severe disease. Notably, these variations were not significantly influenced by age, BMI, or geographical latitude, and CPAP therapy did not increase serum 25(OH)D concentrations [18].

In the current study, 25(OH)D levels were negatively correlated with indoor occupational activity. The results of the 5th Korea National Health and Nutrition Examination Survey (KNHANES 2010–2012), with 5409 participants, revealed that working conditions and 25(OH)D deficiency were significantly associated among male shift workers, office workers, and permanent workers, while no such association was observed among female participants [28]. According to a meta-analysis of 71 studies, shift workers (80%) and indoor workers (78%) had higher rates of 25(OH)D deficiency than outdoor workers (48%). Furthermore, compared to outdoor workers, indoor workers had considerably lower 25(OH)D levels (*p* < 0.0001) [15]. Overall, 25(OH)D concentrations reported in that meta-analysis were higher than those in our population, likely reflecting the added impact of OSAS as an additional risk factor. These findings are supported by another systematic review showing that shift workers and workers in indoor environments are the occupational groups most susceptible to 25(OH)D insufficiency [16].

As expected, subjects working indoors are less exposed to sunlight. Of note, glass used in buildings absorbs all UVB radiation, preventing cutaneous 25(OH)D synthesis [29]. Furthermore, indoor workers with traditional working hours are typically exposed to sunlight only during early morning or late afternoon hours, when UVB intensity is insufficient for optimal 25(OH)D production [29]. This restriction may be affected by shift work that involves evening or nocturnal shifts. Overall, the available evidence remains inconclusive with respect to the impact of occupational characteristics (e.g., shift work, indoor work, work activities) on 25(OH)D status, suggesting that sunlight exposure alone does not fully explain 25(OH)D deficiency in working populations. Nevertheless, there are studies that support this, reporting comparable serum 25(OH)D concentrations between indoor and outdoor workers [30,31].

From the opposite perspective, several cardinal features of OSAS may also adversely affect 25(OH)D levels, e.g., obesity. It has been demonstrated that in healthy individuals, circulating 25(OH)D concentrations are inversely correlated with higher body fat percentages [32]. Another study reported that obese individuals tend to spend less time in outdoor activities, including sun exposure, a behaviour that has been associated with higher body fat percentage and BMI [33]. 25(OH)D is stored in adipose tissue [30,31]. The capacity of adipose tissue to locally metabolize 25(OH)D is shifted towards decreased synthesis and increased catabolism in obese individuals [34]. As a result of sequestration within adipose tissue and reduced systemic bioavailability, oral 25(OH)D supplementation results in smaller increases in serum 25(OH)D levels in obese compared to non-obese individuals [35,36]. Moreover, the volumetric dilution of 25(OH)D within an expanded adipose tissue mass, regardless of whether it is derived from dietary intake or cutaneous synthesis, may contribute to hypovitaminosis in obese individuals [37]. Excessive daytime sleepiness, another feature of OSAS as a result of sleep fragmentation, may also indirectly lower serum 25(OH)D levels by limiting outdoor activities and sun exposure [19]. As mentioned previously, vitamin D has been shown to play a role in the regulation of the sleep–wake cycle by modulating melatonin synthesis and secretion through receptors and metabolizing enzymes located in brain regions that are involved in circadian regulation [20]. However, in the current study neither excessive daytime sleepiness nor increased BMI were predictors of 25(OH)D insufficiency.

The following limitations of this study should be acknowledged: First, relatively small sample size. Larger studies are necessary to better evaluate the effect of occupational setting on 25(OH)D levels in OSAS populations. Second, important information, such as skin pigmentation and dietary habits of the participants, was not evaluated. Still, the study was carried out over a restricted 12-month timeframe in a homogeneous cohort of Caucasian participants residing in the same geographic area, with broadly similar dietary and clothing behaviours, which likely minimized interindividual variability. Third, 25(OH)D levels were not adjusted for seasonality. However, blood samples were collected evenly across all months, and clustering of measurements was avoided, particularly during winter or summer, thus minimizing the effect of seasonal variability of 25(OH)D levels. It should also be noted that wintertime serum 25(OH)D concentrations are partly related to summertime levels due to storage of 25(OH)D in skeletal muscle and its subsequent release into the circulation [38,39]. Fourth, this study primarily included middle-aged participants; therefore, caution should be exercised when extrapolating these findings to older OSAS patients, as both OSAS prevalence and 25(OH)D insufficiency increase with age [40,41]. Finally, detailed cardiovascular assessments were not performed, which may be relevant given the established associations between 25(OH)D status and vascular morbidity [36,37].

## 5. Conclusions

In conclusion, indoor working is associated with lower serum 25(OH)D levels in patients with OSAS. Therefore, the occupational setting should be taken into account when evaluating 25(OH)D status in individuals with OSAS.

## Figures and Tables

**Table 1:** Demographic and clinical characteristics of the study population.

	Overall Sample (n = 189)	OSAS Patients (n = 129)	Non-Apneic Controls (n = 60)	*p* Value (OSAS vs. Controls)
**Sex (males/females)**	155/34	110/19	45/15	0.09
**Age (years)**	48 ± 10	48 ± 9	46 ± 11	0.138
**OSAS severity (n)**	–	Mild: 12 Moderate: 17 Severe: 100	–	–
**Serum 25(OH)D (ng/mL)**	22 ± 11	19 ± 9	28 ± 11	<0.001

**Table 2:** Demographic and clinical characteristics of the study groups.

	Group A (Non-OSAS Subjects Working Indoors) (n = 30)	Group B (Non-OSAS Subjects Working Outdoors) (n = 30)	Group C (OSAS Patients Working Indoors) (n = 64)	Group D (OSAS Patients Working Outdoors) (n = 65)	*p*
**Gender (male/female)**	21/9	24/6	55/9	55/10	0.26
**Age (years)**	48.3 ± 11.3	43.6 ± 9.7	47.5 ± 9.4	48.9 ± 9.1	0.09
**BMI (kg/m^2^)**	32.3 ± 7	31.9 ± 6.6	35.6 ± 7.6	37.8 ± 7.4 *^, #^	0.001
**Neck circumference (cm)**	41.9 ± 3.9	39.2 ± 4.6	44.1 ± 3.8 ^##^	45.7 ± 3.6 **^, ##^	<0.001
**Waist circumference (cm)**	108.3 ± 18.9	107.3 ± 15.4	121.3 ± 15.3 *^, #^	125.4 ± 14 **^, ##^	<0.001
**Hip circumference (cm)**	110 ± 20	112.8 ± 13	119.6 ± 14.8	120.8 ± 17.7	0.052
**WHR**	0.98 ± 0.09	0.95 ± 0.07	1.02 ± 0.07	1.05 ± 0.13 *^, ##^	0.001
**Married (%)**	80%	83%	78.1%	78.5%	0.94
**More than elementary education (%)**	100%	93.3%	100%	89.2% *^,^ ^	0.02
**Smoking (%)**	40%	40%	35.9%	36.9%	0.97

Abbreviations: BMI: body mass index, WHR: waist to hip ratio. *: *p* < 0.05 compared with non-OSAS subjects working indoors; **: *p* < 0.001 compared with non-OSAS subjects working indoors; ^#^: *p* < 0.05 compared with non-OSAS subjects working outdoors; ^##^: *p* < 0.001 compared with non-OSAS subjects working outdoors; ^: *p* < 0.05 compared with OSAS patients working outdoors.

**Table 3:** Comparison of sleep parameters between groups.

	Group A (Non-OSAS Subjects Working Indoors) (n = 30)	Group B (Non-OSAS Subjects Working Outdoors) (n = 30)	Group C (OSAS Patients Working Indoors) (n = 64)	Group D (OSAS Patients Working Outdoors) (n = 65)	*p*
**TST (min)**	302.1 ± 67	317.6 ± 52.5	327 ± 64.9	316.9 ± 51.1	0.30
**N1 (% TST)**	9.7 (5.2–16)	7.4 (4–16.5)	8.8 (4.4–15.8)	10 (4.1–18.5)	0.65
**N2 (% TST)**	63.9 ± 12.5	66 ± 9.9	70 ± 15.8	70.3 ± 16.2	0.13
**N3 (% TST)**	12.9 (5.6–24.1)	13.1 (5–21.9)	5.1 (0.4–15) *^, #^	4.7 (0.1–13.8) *^, #^	<0.001
**REM (% TST)**	9.9 (3.9–14)	9.6 (3.1–14.5)	6.5 (0.5–11)	5.4 (2–10.8)	0.11
**AHI (events/h)**	3.4 (1.7–4.5)	3.3 (1–4.4)	59.9 (33.5–72.9) **^, ##^	55.2 (25.9–73) **^, ##^	<0.001
**Aver SaO_2_ (%)**	94.2 (92.8–94.7)	94.7 (93–96.1)	92 (89–93) **^, ##^	91(88.4–92.9) **^, ##^	<0.001
**Min SaO_2_ (%)**	85.7 ± 5	84.1 ± 16.4	70.1 ± 11.3 **^, #^	70.3 ± 10.1 **^, #^	<0.001
**T < 90% (%)**	0 (0–1.8)	0.2 (0–2.6)	24.6 (8.7–51.8) **^, ##^	33.8 (10.2–59.8) **^, ##^	<0.001
**Arousal index**	15.2 ± 8.8	15.9 ± 6.3	33.5 ± 20.2 **^, ##^	35.2 ± 22.9 **^, ##^	<0.001
**Sleep efficiency (%)**	83 (72–90)	85.5 (75–93.4)	89.8 (82.3–93.3) *	87.1 (80.3–92.7) *	0.02
**ESS score**	6.4 ± 4.2	9.2 ± 4.6	11.8 ± 5.1 **	12 ± 5.5 **	<0.001

Abbreviations: AHI: Apnoea–Hypopnoea Index, Aver SaO_2_: average oxyhaemoglobin saturation, ESS: Epworth sleepiness scale, Min SaO_2_: minimum oxyhaemoglobin saturation, N1: sleep stage 1, N2: sleep stage 2, N3: sleep stage 3, REM: rapid eye movement, TST: total sleep time, T < 90%: time with oxyhaemoglobin saturation < 90%. *: *p* < 0.05 compared with non-OSAS subjects working indoors; **: *p* < 0.001 compared with non-OSAS subjects working indoors; ^#^: *p* < 0.05 compared with non-OSAS subjects working outdoors; ^##^: *p* < 0.001 compared with non-OSAS subjects working outdoors.

**Table 4:** Comparison of different laboratory results between groups.

	Group A (Non-OSAS Subjects Working Indoors) (n = 30)	Group B (Non-OSAS Subjects Working Outdoors) (n = 30)	Group C (OSAS Patients Working Indoors) (n = 64)	Group D (OSAS Patients Working Outdoors) (n = 65)	*p*
**FEV_1_ (% predicted)**	102.9 ± 15.4	101.4 ± 23.4	93.2 ± 16	91.9 ± 17 *	0.03
**FVC (% predicted)**	99.3 ± 11.7	99.2 ± 21.1	88.8 ± 15.6 *	89.2 ± 18.6 *	0.01
**pO_2_ (mmHg)**	85.1 ± 9.6	90.4 ± 19.6	80.3 ± 8.7	79.4 ± 8.8 *^, #^	<0.001
**pCO_2_ (mmHg)**	40.9 ± 2.4	40 ± 4.8	41.4 ± 4.2	42.6 ± 5.3	0.06
**Glucose (mg/dL)**	97 (81–113.5)	90 (80–112.5)	96.5 (91.5–110)	108 (84–137)	0.08
**Creatinine (mg/dL)**	0.93 ± 0.16	0.92 ± 0.28	0.93 ± 0.16	0.91 ± 0.18	0.96
**SGOT (mg/dL)**	25 ± 7.2	24.7 ± 6.9	22.9 ± 6.9	22.3 ± 5.8	0.37
**SGPT (mg/dL)**	27 (18.5–41)	24 (18–32.5)	26.5 (21–35.3)	29 (18–48)	0.81
**Cholesterol (mg/dL)**	209.5 ± 43.9	189.6 ± 39	209.3 ± 47	214.2 ± 41.3	0.28
**Triglycerides (mg/dL)**	154.5 (99.3–204.8)	123.5 (75.8–167.3)	159 (106–198)	163.5 (111–220.3)	0.37
**LDL-C (mg/dL)**	132.4 ± 33.2	112.6 ± 35.6	126.4 ± 36.6	124.3 ± 33.4	0.36
**HDL-C (mg/dL)**	47.4 ± 14.9	50.8 ± 13.3	47.2 ± 12.4	48.8 ± 13	0.78
**25(OH)D (ng/mL)**	22.9 ± 6.7	32.2 ± 13 *	15.2 ± 7.6 **^, ##^	22.9 ± 9.3 ^#^	<0.001

Abbreviations: ALT: alanine aminotransferase, AST: aspartate aminotransferase, FEV_1_: forced expiratory volume in 1st sec, FVC: forced vital capacity, HDL-C: high-density lipoprotein cholesterol, LDL-C: low-density lipoprotein cholesterol, pCO_2_: carbon dioxide partial pressure, pO_2_: oxygen partial pressure, 25(OH)D: 25-hydroxyvitamin D. *: *p* < 0.05 compared with non-OSAS subjects working indoors; **: *p* < 0.001 compared with non-OSAS subjects working indoors; ^#^: *p* < 0.05 compared with non-OSAS subjects working outdoors; ^##^: *p* < 0.001 compared with non-OSAS subjects working outdoors.

**Table 5:** Serum 25(OH)D levels according to occupation and OSAS status.

Groups	n	Serum 25(OH)D (ng/mL)
**Group A: Non-OSAS subjects working indoors**	30	22.9 ± 6.7 ^##^
**Group B: Non-OSAS subjects working outdoors**	30	32.2 ± 13 *^, ##,^ ^^
**Group C: OSAS patients working indoors**	64	15.2 ± 7.6
**Group D: OSAS patients working outdoors**	65	22.9 ± 9.3

*: *p* < 0.05 compared with non-OSAS subjects working indoors; ^##^: *p* < 0.001 compared with OSAS subjects working indoors; ^^: *p* < 0.001 compared with OSAS patients working outdoors.

## Data Availability

Data available upon request due to restrictions.

## Abbreviations

The following abbreviations are used in this manuscript:

25(OH)D	25-hydroxy-vitamin D
AHI	Apnea-Hypopnea Index
ALT	Alanine Aminotransferase
AST	Aspartate Aminotransferase
Aver SaO_2_	Average Oxyhaemoglobin Saturation
BMI	Body Mass Index
CRP	C-Reactive Protein
ESS	Epworth Sleepiness Scale
FEV_1_	Forced Expiratory Volume in 1st sec
FVC	Forced Vital Capacity
HDL-C	High-Density Lipoprotein Cholesterol
LDL-C	Low-Density Lipoprotein Cholesterol
Min SaO_2_	Minimum Oxyhaemoglobin Saturation
N1	Sleep Stage 1
N2	Sleep Stage 2
N3	Sleep Stage 3
OSAS	Obstructive Sleep Apnea Syndrome
pCO_2_	Carbon dioxide Partial Pressure
pO2	Oxygen Partial Pressure
PSG	Polysomnography
REM	Rapid Eye Movement
T < 90%	Time with Oxyhaemoglobin Saturation <90%
TST	Total Sleep Time
